# Prognostic potential of liver injury in patients with dilated cardiomyopathy: a retrospective study

**DOI:** 10.1186/s40001-022-00876-9

**Published:** 2022-11-08

**Authors:** Hai-Yan Wang, Yuan Huang, Xiao-Zhen Chen, Zi-Lan Zhang, Chun Gui

**Affiliations:** 1 Department of Cardiology, The First Affiliated Hospital of Guangxi Medical University, No 6, Shuangyong Road, Nanning, 530021 Guangxi China; 2 Guangxi Key Laboratory Base of Precision Medicine in Cardio-Cerebrovascular Diseases Control and Prevention, No 6, Shuangyong Road, Nanning, 530021 Guangxi China; 3 Guangxi Clinical Research Center for Cardio-Cerebrovascular Diseases, No 6, Shuangyong Road, Nanning, 530021 Guangxi China

**Keywords:** Dilated cardiomyopathy, Liver injury, Risk factor, Prognosis, Mortality

## Abstract

**Background:**

Liver injury (LI) has been frequently observed in patients with dilated cardiomyopathy (DCM), whereas its prognostic value remains blurry. We attempted to appraise the prognostic effect of LI in patients with DCM.

**Methods:**

This retrospective study included 523 patients with DCM. LI was defined as a threefold increase in aspartate transaminase (≥ 135 U/L) or alanine transaminase (≥ 180 U/L) or a twofold increase in total bilirubin (≥ 41 umol/L) during hospitalization. The population was segmented into non-liver injury (NLI) group and LI group based on liver function test data. To balance differences in covariates at baseline, 1:1 propensity score matching (PSM) was performed.

**Results:**

Patients with LI had lower survival rate, compared with those with NLI (44.6% vs. 73.8%, *P* < 0.001). Similar results were also found in age (age > 50, 39.6% vs. 70.9%, *P* < 0.001; age ≤ 50, 51.3% vs. 79.5%, *P* < 0.001) and gender stratified analysis (male, 46.2% vs. 74.4%, *P* < 0.001; female 35.7% vs. 72.0%, *P* = 0.001). After PSM, the survival rate of patients with LI remained lower than those with NLI (44.6% vs. 64.1%, *P* = 0.019). Multivariable Cox regression analysis manifested that LI (hazard ratio [HR]: 1.692, 95% confidence interval [CI] 1.194–2.398, *P* = 0.003; HR: 1.675, 95% CI 1.078–2.604, *P* = 0.022, respectively) showed potent predictive effect on all-cause mortality in patients with DCM, both before and after PSM.

**Conclusions:**

The occurrence of LI herald adverse outcomes in patients with DCM and attention to LI may be conducive to risk stratification and management.

**Supplementary Information:**

The online version contains supplementary material available at 10.1186/s40001-022-00876-9.

## Background

As early as 1930, N. Jolliffe reported a phenomenon of heart failure (HF) accompanied by abnormal liver function (ALF), which predominantly referred to the elevation of bilirubin [1]. Since then, many people have devoted themselves to researching this phenomenon and disclosed that liver function indicators, such as alkaline phosphatase, γ-glutamyl-transpeptidase, transaminase and lactate dehydrogenase, not just bilirubin, may be elevated [2]. A number of recent studies identified that ALF was prevalent in acute and chronic HF and other critically ill patients, and presented unignorable prognostic value [3–7]. Nevertheless, the contribution of liver function indicators to prognosis has been mixed in numerous studies [4, 8–13]. For one thing, a post-hoc analysis of the EVEREST trial for HF with reduced ejection fraction found that reduced serum albumin (ALB) and elevated total bilirubin (TB) were associated with adverse outcomes, while aspartate transaminase (AST) and alanine transaminase (ALT) were not [13]. For another, elevated AST and ALT predicted a poor prognosis of 180 days in the PROTECT study on acute HF, excluding patients with TB > 3 mg/dL or ALB < 2.8 mg/dL [4]. Despite the difference in the baseline characteristics, and inclusion and exclusion criteria were the reason for the diversity in the results, fluctuations of ALF on prognosis arouse confusion and controversy.

Of note, ALF is not entirely equivalent to liver injury (LI), that is, dysfunction of bilirubin metabolism and damage of hepatocytes [3], similar to the relationship between abnormal kidney function and kidney injury [14]. The elevation of AST and ALT is not only seen in liver diseases, but also in myopathic diseases. Similarly, the elevation of TB can also occur in extrahepatic diseases [15]. Compared with ALF, LI has clearer significance and more practical clinical utility [16]. In addition, the absolute values of AST and ALT are weakly associated with the severity or extent of LI, and their influence on prognosis is not reliable [17]. What is more, LI is a comprehensive assessment result, reflects the status of liver function and is convenient for risk stratification. Consequently, we suspected that LI may have a more dependable and robust prognostic effect than a single indicator of liver function. In a word, the impact of LI on the prognosis of patients with dilated cardiomyopathy (DCM) was blurry. We were dedicated to delineating the role of LI in patients with DCM.

## Materials and methods

### Study design

This retrospective study was performed at the First Affiliated Hospital of Guangxi Medical University and included 523 hospitalized DCM patients with HF admitted from August 2012 to May 2020. DCM was defined as left ventricular end-diastolic dimension (LVEDD) > 55 mm for males or > 50 mm for females and left ventricular ejection fraction (LVEF) < 45%, which cannot be explained by hypertension or coronary artery disease, and valvular or congenital heart disease were absent. The diagnosis of dilated cardiomyopathy strictly followed Chinese guidelines. LI was defined as a threefold increase in AST (≥ 135 U/L) or ALT (≥ 180 U/L) or a twofold increase in TB (≥ 41 umol/L) at any time during hospitalization [18]. Inclusion criteria were: (1) the discharge diagnosis was DCM; (2) age ≥ 18 years; (3) New York Heart Association (NYHA) class ≥ II; (4) patients admitted to our hospital for the first time. Our hospital is a regional medical center. To maintain the consistency of the characteristics of the enrolled patients, we included only patients who were discharged after comprehensive evaluation and treatment for the first time in our hospital.

Exclusion criteria were: (1) chronic liver disease; (2) drug-induced liver injury; (3) acute or chronic biliary tract disease; (4) severe inflammatory or infectious diseases; (5) cardiopulmonary resuscitation; (6) new or old myocardial infarction; (7) coronary revascularization; (8) coronary artery disease complicated with myocardial ischemia; (9) acute myocarditis; (10) chronic kidney disease 5 period or need for hemodialysis; (11) heart transplantation; (12) death in hospital; (13) liver function test and cardiac ultrasound data missing; (14) traumatic patients.

All patients underwent physical examinations, blood laboratory tests, 12 lead electrocardiogram and echocardiography during the duration of hospital stay. Medical history collection, physical examinations, 12 lead electrocardiogram and medical records were finished within 8 h of admission. Blood tests of all patients were performed in our central laboratory. Cardiac ultrasonography was implemented by experienced sonographers. Echocardiographic parameters were measured according to the guidelines of the current year [19, 20], and LVEF was measured using the M-mode or modified Simpson’s method, as appropriate, and calculated using the following formula: [EDV (end-diastolic volume) − ESV (end-systolic volume)]/EDV. The values of the hematological indicators tested in the laboratory at admission were recorded and analyzed. Estimated glomerular filtration rate (eGFR) was calculated according to the modified formula suitable for Chinese [21]. Sixteen patients or families refused to participate in this retrospective study. The primary endpoint confirmed by medical records and telephone follow-up was all-cause mortality, and there was an 8.4% loss to follow-up in our cohort. Given the retrospective study design, it was ratified by the Human Research Ethics Committee of the First Affiliated Hospital of Guangxi Medical University [NO.2022-KY-E-(280)], and informed consent was not required. The procedures had abided by the Declaration of Helsinki.

### Statistical analysis

Patients were discriminated into non-liver injury (NLI) group and LI group based on liver function test data. Statistical description of quantitative variables was displayed as the mean ± standard deviation or median (interquartile) according to distribution pattern. Classification variables were statistically described in number (percentage). For comparison of quantitative variables, *t* test or Wilcoxon rank sum test was used between the two groups, as appropriate. Qualitative data were compared using Chi-square test or Fisher exact test. Ordinal data were compared using Wilcoxon rank sum test. The significance comparison of Kaplan–Meier survival plots was tested by log-rank between the two groups. The initial timepoint for survival analysis was the day of discharge. Schoenfeld residuals were computed to test proportional hazard assumption. Relevant parameters for risk of the primary endpoint were appraised by Univariable Cox regression analysis. Subsequently, parameters with *P* < 0.05 were included in Multivariable Cox regression analysis, and were screened using the “forward” option. To balance differences in covariates at baseline between groups and enhance the reliability of results, propensity score matching (PSM) was performed. These covariates involved in age, sex, diabetes mellitus, NYHA class, systolic blood pressure (SBP), ALB, blood–urea–nitrogen (BUN), N-terminal pro-B-type natriuretic peptide (NT-proBNP), left atrium diameter (LAD), and renin–angiotensin system (RAS) inhibitor. Considering the ratio of two groups of patients, a 1:1 nearest-neighbor matching was implemented. All statistical analysis was executed by SPSS 23.0 and R 4.2.0. All tests were two-sided. The statistical significance was determined by *P* < 0.05.

## Results

### Baseline characteristics

Demographic variables, clinical and behavioral features of the population were exhibited in Table 1. 76.9% of all patients were male, and the median age of the enrolled patients was 55.0 (46.0–64.0) years. Some of the patients had a history of alcohol (43.8%) and tobacco (44.9%) use. The proportion of patients with NLI and LI were 82.4% (431/523) and 17.6% (92/523), respectively. Compared with the LI group, patients with NLI were older, had higher SBP and ALB, lower BUN and NT-proBNP level, lower proportion of NYHA IV, smaller LAD. In addition, those with NLI were less likely to be male and have diabetes mellitus. RAS inhibitor use was more common in the NLI group. Higher NYHA classification was associated with a higher proportion of patients with LI (NYHA II 5.6%, NYHA III 16.0%, NYHA IV 27.6%).

**Table 1:** Baseline characteristics of patients categorized by liver injury

Characteristics	All patients (*n* = 523)	Non-liver injury (*n* = 431)	Liver injury (*n* = 92)	*P* value
Age (years)	55.0 (46.0–64.0)	56.0 (46.0–65.0)	51.0 (40.8–59.0)	< 0.001
Male sex, *n* (%)	402 (76.9)	324 (75.2)	78 (84.8)	0.047
Smokers, *n* (%)	235 (44.9)	186 (43.2)	49 (53.3)	0.077
Drinkers, *n* (%)	229 (43.8)	183 (42.5)	46 (50.0)	0.186
BMI (kg/m^2^)	23.1 (20.6–26.0)	23.3 (20.8–26.0)	22.7 (19.9–25.0)	0.111
Comorbidity, *n* (%)				
Acute heart failure	406 (77.6)	329 (76.3)	77 (83.7)	0.124
Coronary artery disease	30 (5.7)	28 (6.5)	2 (2.2)	0.106
Hypertension	80 (15.3)	71 (16.5)	9 (9.8)	0.106
Diabetes mellitus	76 (14.5)	56 (13.0)	20 (21.7)	0.031
Atrial fibrillation	97 (18.5)	77 (17.9)	20 (21.7)	0.386
NYHA class, *n* (%)				< 0.001
II	125 (23.9)	118 (27.4)	7 (7.6)	
III	213 (40.7)	179 (41.5)	34 (37.0)	
IV	185 (35.4)	134 (31.1)	51 (55.4)	
SBP (mmHg)	114.0 (102.0–126.0)	116.0 (103.0–128.0)	106.0 (97.0–117.0)	< 0.001
DBP (mmHg)	75.0 (68.0–86.0)	76.0 (66.0–86.0)	75.0 (69.0–84.0)	0.788
Laboratory data				
Red blood cell (× 10^12^/L)	4.73 (4.34–5.17)	4.73 (4.31–5.13)	4.91 (4.47–5.36)	0.083
Hemoglobin (g/L)	136.8 (126.6–147.7)	136.6 (126.0–147.1)	138.1 (128.2–150.5)	0.232
Albumin (g/L)	38.50 ± 4.62	38.71 ± 4.65	37.55 ± 4.33	0.029
AST (U/L)	31.0 (23.0–43.0)	29.0 (23.0–39.0)	47.0 (31.3–137.5)	< 0.001
ALT (U/L)	31.0 (19.0–51.0)	30.0 (18.0–45.0)	53.5 (29.3–180.0)	< 0.001
Total bilirubin (μmol/L)	17.0 (11.0–28.1)	14.2 (9.9–21.6)	46.1 (33.5–56.4)	< 0.001
BUN (mmol/L)	6.90 (5.42–8.77)	6.64 (5.35–8.52)	8.02 (5.83–11.14)	< 0.001
eGFR (mL/min/1.73 m^2^)	91.6 (74.6–110.5)	91.4 (75.7–109.8)	92.3 (72.2–118.8)	0.705
NT-proBNP (pg/Ml)	3723 (1766–7065)	3301 (1609–6481)	6481 (2689–12,152)	< 0.001
QRS duration (ms)	106.0 (98.0–122.0)	106.0 (96.0–122.0)	107.0 (98.5–118.0)	0.687
Cardiac ultrasound data				
LAD (mm)	46.0 (42.0–51.0)	46.0 (41.0–50.0)	49.5 (44.0–53.0)	< 0.001
LVEDD (mm)	70.0 (64.0–76.0)	69.0 (64.0–76.0)	72.0 (65.0–78.0)	0.118
LVEF (%)	33.0 (27.0–38.0)	33.0 (27.0–38.0)	31.0 (25.0–38.0)	0.281
Drug therapy, *n* (%)				
RAS inhibitor	460 (88.0)	389 (90.3)	71 (77.2)	< 0.001
ACEI/ARB	408 (78.0)	343 (79.6)	65 (70.7)	0.060
ARNI	52 (9.9)	46 (10.7)	6 (6.5)	0.227
Beta-blocker	471 (90.1)	385 (89.3)	86 (93.5)	0.227
Diuretic	520 (99.4)	428 (99.3)	92 (100.0)	1.000
Spironolactone	501 (95.8)	416 (96.5)	85 (92.4)	0.086
Digoxin	415 (79.3)	340 (78.9)	75 (81.5)	0.571
ICD/CRT, *n* (%)	23 (4.4)	19 (4.4)	4 (4.3)	1.000

Data are displayed as the mean ± SD (standard deviation), or median (interquartile range), or number (percentage). BMI data were missing in 9 cases. Electrocardiogram were missing in 42 cases

BMI: body mass index; NYHA: New York Heart Association; SBP: systolic blood pressure; DBP: diastolic blood pressure; AST: aspartate transaminase; ALT: alanine transaminase; BUN: blood–urea–nitrogen; eGFR: estimated glomerular filtration rate; NT-proBNP: N-terminal pro-B-type natriuretic peptide; LAD: left atrium diameter; LVEDD: left ventricular end-diastolic dimension; LVEF: left ventricular ejection fraction; RAS: renin–angiotensin system; ACEI: angiotensin-converting enzyme inhibitor; ARB: angiotensin receptor blocker; ARNI: angiotensin receptor neprilysin inhibitor; ICD: implantable cardioverter defibrillator; CRT: cardiac resynchronization therapy

After PSM, the number of DCM patients in the LI and NLI groups was equivalent (92 vs. 92) (Table 2). Except for differences in ATS, ALT, and TB, the characteristics of baseline variables were equalized between the LI and NLI groups (all *P* > 0.05).

**Table 2:** Baseline characteristics of patients categorized by liver injury after propensity score matching

Characteristics	All patients (*n* = 184)	Non-liver injury (*n* = 92)	Liver injury (*n* = 92)	*P* value
Age (years)	51.0 (38.0–58.0)	49.0 (37.3–57.8)	51.0 (40.8–59.0)	0.434
Male sex, *n* (%)	149 (81.0)	71 (77.2)	78 (84.8)	0.189
Smokers, *n* (%)	97 (52.7)	48 (52.2)	49 (53.3)	0.883
Drinkers, *n* (%)	86 (46.7)	40 (43.5)	46 (50.0)	0.375
BMI (kg/m^2^)	22.7 (20.0–25.9)	23.0 (20.2–26.5)	22.7 (19.9–25.0)	0.480
Comorbidity, *n* (%)				
Acute heart failure	157 (85.3)	80 (87.0)	77 (83.7)	0.532
Coronary artery disease	6 (3.3)	4 (4.3)	2 (2.2)	0.682
Hypertension	20 (10.9)	11 (12.0)	9 (9.8)	0.636
Diabetes mellitus	40 (21.7)	20 (21.7)	20 (21.7)	1.000
Atrial fibrillation	39 (21.2)	19 (20.7)	20 (21.7)	0.857
NYHA class, *n* (%)				0.596
II	14 (7.6)	7 (7.6)	7 (7.6)	
III	72 (39.1)	38 (41.3)	34 (37.0)	
IV	98 (53.3)	47 (51.1)	51 (55.4)	
SBP (mmHg)	106.0 (97.0–117.8)	106.0 (98.0–118.0)	106.0 (97.0–117.0)	0.666
DBP (mmHg)	74.0 (66.0–84.0)	71.0 (64.0–84.3)	75.0 (69.0–84.0)	0.111
Laboratory data				
Red blood cell (× 10^12^/L)	4.75 (4.29–5.23)	4.69 (4.19–5.03)	4.91 (4.47–5.36)	0.070
Hemoglobin (g/L)	137.0 (128.4–149.9)	137.0 (128.9–149.0)	138.1 (128.2–150.5)	0.801
Albumin (g/L)	37.1 ± 4.46	36.71 ± 4.56	37.55 ± 4.33	0.202
AST (U/L)	37.0 (28.0–62.0)	33.0 (23.0–43.0)	47.0 (31.3–137.5)	< 0.001
ALT (U/L)	38.5 (24.0–88.8)	31.0 (20.3–48.8)	53.5 (29.3–180.0)	< 0.001
Total bilirubin (μmol/L)	28.6 (16.8–46.2)	17.7 (10.9–23.8)	46.1 (33.5–56.4)	< 0.001
BUN (mmol/L)	7.73 (5.65–10.69)	7.38 (5.53–9.66)	8.02 (5.83–11.14)	0.123
eGFR (mL/min/1.73 m^2^)	89.9 (70.9–114.5)	88.7 (70.4–112.8)	92.3 (72.2–118.8)	0.484
NT-proBNP (pg/mL)	6051 (2881–10,139)	5779 (2916–9574)	6481 (2689–12,152)	0.478
QRS duration (ms)	106.0 (98.0–118.0)	106.0 (96.0–121.0)	107.0 (98.5–118.0)	0.443
Cardiac ultrasound data				
LAD (mm)	48.0 (43.3–53.0)	46.5 (43.0–52.0)	49.5 (44.0–53.0)	0.173
LVEDD (mm)	71.0 (65.0–77.0)	70.5 (66.0–76.0)	72.0 (65.0–78.0)	0.546
LVEF (%)	31.0 (25.0–37.0)	31.0 (24.6–37.0)	31.0 (25.0–38.0)	0.816
Drug therapy, *n* (%)				
RAS inhibitor	144 (78.3)	73 (79.3)	71 (77.2)	0.721
Beta-blocker	169 (91.8)	83 (90.2)	86 (93.5)	0.419
Diuretic	184 (100.0)	92 (100.0)	92 (100.0)	1.000
Spironolactone	174 (94.6)	89 (96.7)	85 (92.4)	0.193
Digoxin	155 (84.2)	80 (87.0)	75 (81.5)	0.312
ICD/CRT, *n* (%)	8 (4.3)	4 (4.3)	4 (4.3)	1.000

### Primary endpoint

**Fig. 1:**
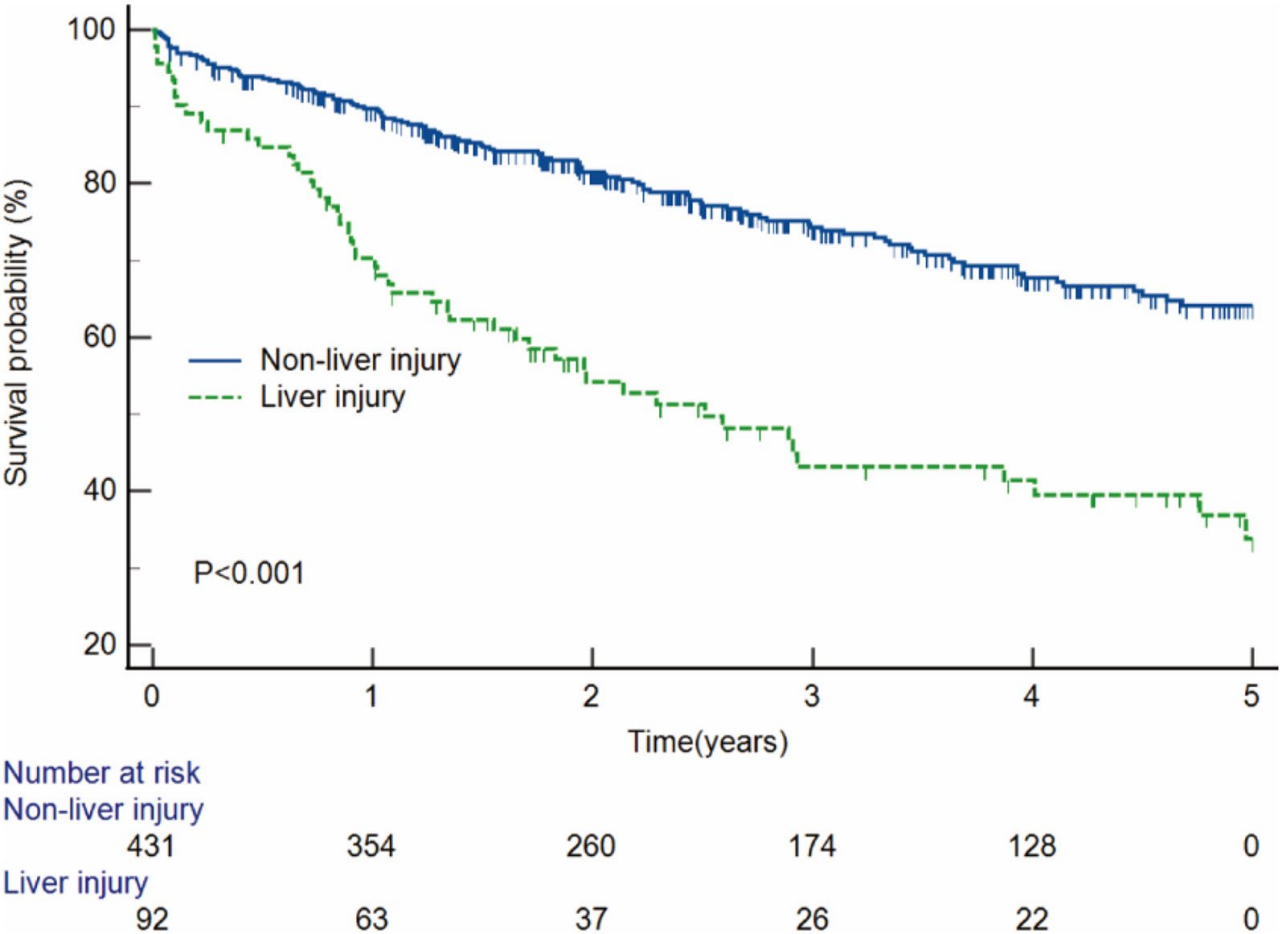
Kaplan–Meier curves estimated the occurrence of the primary outcome in patients with and without liver injury

**Fig. 2:**
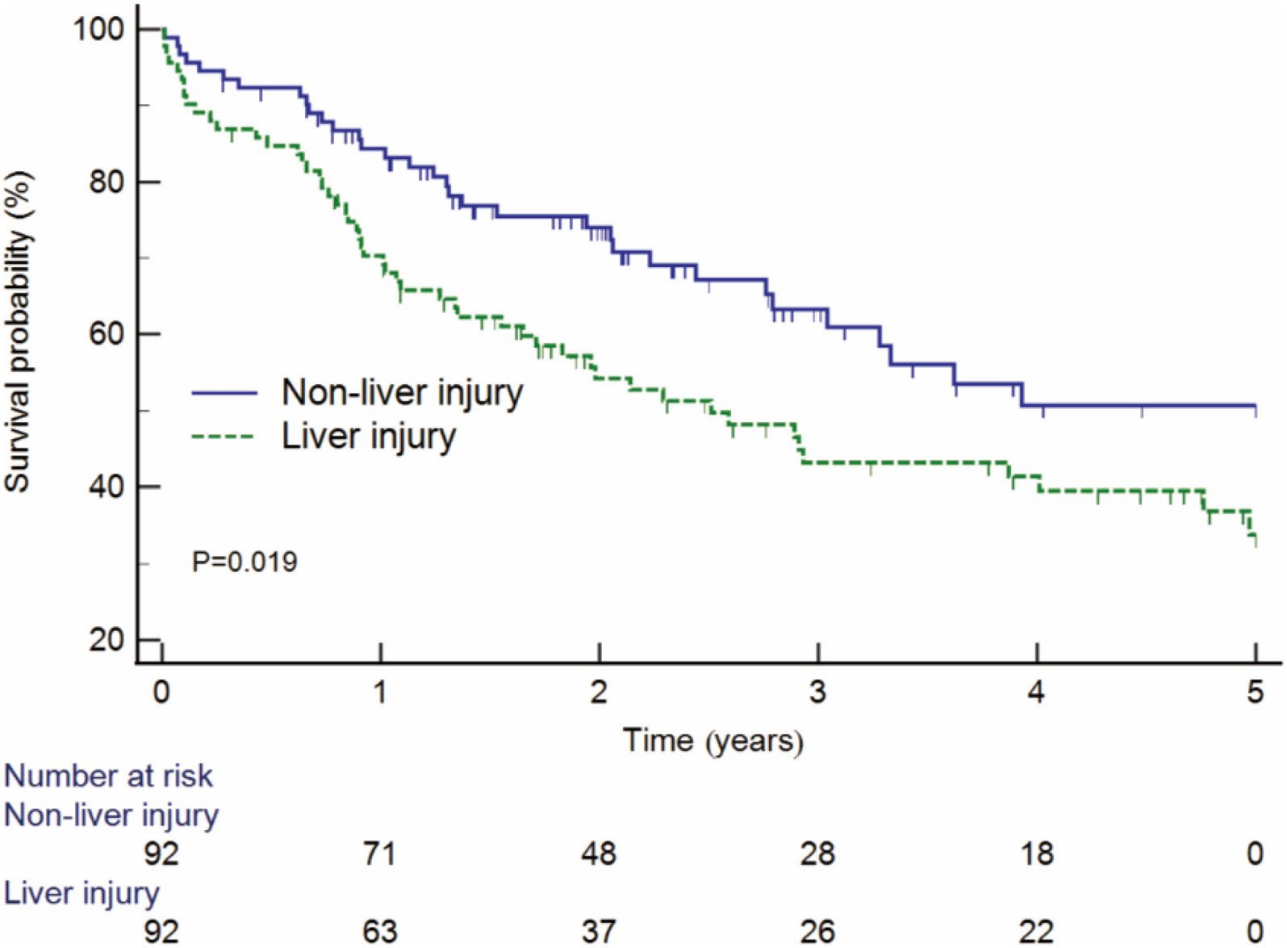
Kaplan–Meier curves estimated the occurrence of the primary outcome in patients with and without liver injury after propensity score matching

By the end of follow-up at a median time of 2.4 years, 164 of 523 had a primary endpoint. There were 51 cases (55.4%, 51/92) in the LI group and 113 cases (26.2%, 113/431) in the NLI group. The Kapan–Meier survival curves testified that patients with LI had lower survival rate than those with NLI (44.6% vs. 73.8%, *P* < 0.001) (Fig. 1). To confirm the robustness of the results, stratified analysis was performed. The survival rate of patients with LI was lower than that of patients with NLI in different age stratifications (age > 50, 39.6% vs. 70.9%, *P* < 0.001; age ≤ 50, 51.3% vs. 79.5%, *P* < 0.001) (Additional file 1: Fig. S1A, B). Similar results were observed in gender stratified analysis (male, 46.2% vs. 74.4%, *P* < 0.001; female 35.7% vs. 72.0%, *P* = 0.001) (Additional file 1: Fig. S1C, D). The survival rate of patients with LI was also lower than those with NLI in the PSM cohort (44.6% vs. 64.1%, *P* = 0.019) (Fig. 2).

The prediction effect of population characteristics, comorbidities, assessment of cardiac function, hematological indicators, cardiac ultrasound data, and medication and instrument therapy on the primary endpoint are shown in Table 3 by Univariable Cox regression analysis. Body mass index, diabetes mellitus, NYHA class, SBP, BUN, AST, TB, NT-proBNP, LAD, LVEDD, LVEF, RAS inhibitor, and LI showed potent predictive value on the primary endpoint in patients with DCM in the Univariable Cox regression models before baseline adjustment using PSM (all *P* < 0.05). SBP, NT-proBNP, LAD, LVEDD, LVEF, RAS inhibitor and LI played similar prognostic roles in the Univariable Cox regression models after baseline adjustment using PSM (all *P* < 0.05).

**Table 3:** Univariable Cox regression analyses for all-cause mortality in patients with dilated cardiomyopathy

Variable	Before baseline adjustment using PSM			After baseline adjustment using PSM		
	HR	95% CI	*P* value	HR	95% CI	*P* value
Age	1.005	0.993–1.018	0.383	1.006	0.990–1.023	0.435
Male	1.049	0.732–1.503	0.796	1.075	0.624–1.854	0.794
BMI	0.943	0.904–0.984	0.007	0.965	0.914–1.019	0.199
Hypertension	0.789	0.489–1.272	0.331	0.827	0.399–1.714	0.609
Diabetes mellitus	1.525	1.025–2.268	0.037	1.354	0.817–2.242	0.239
Atrial fibrillation	0.988	0.665–1.468	0.953	0.779	0.451–1.343	0.369
NYHA class						
IV/II–III	1.556	1.144–2.118	0.005	1.405	0.909–2.173	0.126
SBP	0.972	0.963–0.982	< 0.001	0.972	0.957–0.987	< 0.001
BUN	1.064	1.029–1.101	< 0.001	1.036	0.997–1.077	0.074
eGFR ≥ 60 mL/min/1.73 m^2^	0.649	0.402–1.047	0.076	0.813	0.450–1.471	0.494
AST	1.002	1.000–1.004	0.021	1.000	0.998–1.003	0.805
ALT	1.001	1.000–1.002	0.168	1.000	0.998–1.001	0.773
Total bilirubin	1.005	1.002–1.008	0.004	1.001	0.997–1.005	0.610
Lg (NT-proBNP)	3.533	2.451–5.093	< 0.001	2.698	1.566–4.649	< 0.001
QRS	1.005	1.000–1.010	0.071	1.006	0.999–1.013	0.096
LAD	1.041	1.021–1.062	< 0.001	1.028	1.001–1.056	0.041
LVEDD	1.044	1.027–1.061	< 0.001	1.036	1.014–1.059	0.001
LVEF < 35%	1.637	1.173–2.283	0.004	2.000	1.228–3.260	0.005
RAS inhibitor	0.548	0.368–0.814	0.003	0.587	0.365–0.943	0.028
Beta-blocker	0.900	0.563–1.438	0.660	0.964	0.464–2.004	0.922
Spironolactone	0.761	0.374–1.549	0.451	0.699	0.283–1.727	0.438
ICD/CRT	0.837	0.393–1.786	0.646	1.137	0.416–3.108	0.802
Liver injury	2.583	1.855–3.597	< 0.001	1.677	1.082–2.599	0.021

**Fig. 3:**
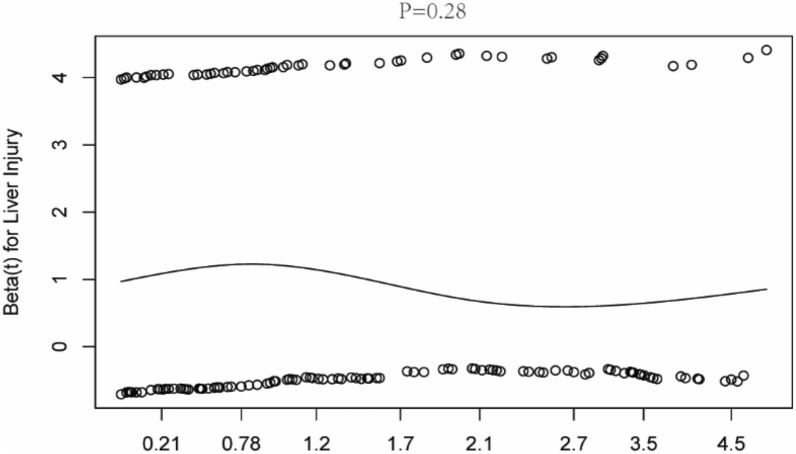
Test of the proportional hazard assumption of Cox regression for liver injury

Schoenfeld residuals were used to verify the relationship between hazard ratio and time. The result proved that proportional hazard assumption was applicable to LI (*P* = 0.28) (Fig. 3). In the Multivariable Cox regression model, LI presented vigorous negative impact on prognosis (hazard ratio [HR]: 1.692, 95% confidence interval [CI] 1.194–2.398, *P* = 0.003; HR: 1.675, 95% CI 1.078–2.604, *P* = 0.022, respectively), both before and after PSM (Table 4).

**Table 4:** Multivariable Cox regression analyses for all-cause mortality in patients with dilated cardiomyopathy

Variable	Before baseline adjustment using PSM			After baseline adjustment using PSM		
	HR	95% CI	*P* value	HR	95% CI	*P* value
SBP	0.976	0.966–0.986	< 0.001	0.973	0.958–0.988	0.001
Lg (NT-proBNP)	3.369	2.279–4.982	< 0.001	2.797	1.586–4.932	< 0.001
LVEDD	1.040	1.022–1.058	< 0.001	1.039	1.014–1.064	0.002
LVEF < 35%		Unselected		1.743	1.054–2.883	0.030
Liver injury	1.692	1.194–2.398	0.003	1.675	1.078–2.604	0.022
AST		Unselected			Unselected	
ALT		Unselected			Unselected	
TB		Unselected			Unselected	

Model was adjusted for BMI, diabetes mellitus, NYHA class, BUN, LAD, LVEF and RAS inhibitor before PSM

Model was adjusted for LAD, and RAS inhibitor after PSM

The risk of death was approximately 1.7 times higher in LI patients than in NLI patients. However, AST, ALT, and TB did not show independent prognostic effects when they were included instead of LI in the multivariable Cox regression model. In addition, after adjusting for confounding factors, SBP (HR: 0.976, 95% CI 0.966–0.986, *P* < 0.001; HR: 0.973, 95% CI 0.958–0.988, *P* = 0.001, respectively), NT-proBNP (HR: 3.369, 95% CI 2.279–4.982, *P* < 0.001; HR: 2.797, 95% CI 1.586–4.932, *P* < 0.001, respectively) and LVEDD (HR: 1.040, 95% CI 1.022–1.058, *P* < 0.001; HR: 1.039, 95% CI 1.014–1.064, *P* = 0.002, respectively) all showed strong independent prognostic value, while LVEF (HR: 1.743, 95% CI 1.054–2.883, *P* = 0.030) was only independently associated with prognosis after PSM.

## Discussion

In the current study, we demonstrated for the first time that the survival rate was markedly distinguished between patients with LI and NLI in DCM, with lower survival rate in the former, which was consistent across stratified studies of different age and gender. In the PSM cohort, the survival rate was still lower in patients with LI. LI emerged powerfully negative effect on survival, regardless of adjustment for potential confounders. After PSM, the same results still hold. Using PSM method, the influence of potential covariates was eliminated, the comparability between the two groups of patients was higher, and the reliability of the results was strengthened. The majority of patients included in previous studies had only slight ALF (liver function indicators < 2–3 times the upper limit of normal) [4, 6, 8, 9, 13], and the value of LI has not been thoroughly affirmed. In clinical setting, there is a significant difference between the treatment and management of patients with slight ALF and LI. Our research focusing on LI is beneficial to clinical work and makes up for the shortcomings of previous studies. This is the first literature on the prognostic potential of LI in DCM, and the results have positive implications for the stratified management of patients with DCM.

When AST, ALT and TB were included as a continuous variable in the final model, they did not show an independent predictive value for prognosis in patients with DCM. The reason for this may be that in this study, the changes of liver function indicators within the normal range or slight increase had no potential clinical implication. Only when LI occurred, the prognostic value of liver function indicators was highlighted. Although previous studies [4, 13] do not entirely support the ideas presented here, the omission of vital measures such as NT-proBNP [4], LAD and LVEDD [13] from the final models may have weakened the applicability of their conclusions to patients with DCM.

There are no epidemiological studies on AFL or LI in outpatients with cardiovascular disease. A reported ALF occurs in 1–4% of asymptomatic patients [15]. We speculated that LI in outpatients with DCM has extremely low incidence and is not easily perceived. Moreover, whether the LI in outpatients with DCM is related to drugs or infectious diseases requires a comprehensive and complex evaluation process. In conclusion, we considered that LI in inpatients compared with LI in outpatients is easier to be found and judged, and has higher application value.

With the early diagnosis and etiological intervention, the development of comprehensive drug therapy and the improvement of device therapy, the survival rate of DCM patients has been considerably improved [22]. During a median follow-up of 2.4 years, approximately 31.4% of patients with DCM had a primary endpoint event in our study, which is apparently extremely high. The high mortality rate can be explained by the following reasons. First, according to the inclusion criteria and Chinese guidelines, patients with NYHA I or LVEF ≥ 45% were not included, who have better cardiac function and a favorable prognosis. Second, the rate of device therapy was low (4.4%). Third, because of the retrospective design, there was an 8.4% loss to follow-up in our cohort.

The liver is supplied by both the portal vein (70%) and the hepatic artery (30%), and its blood flow accounts for about one-fifth to one-quarter of the cardiac output [23]. It is involved in the uptake and transformation of indirect bilirubin as well as the excretion of direct bilirubin. Under the condition of hypoxia, the ability of the liver to uptake, transformation and excretion of bilirubin is weakened, which will inevitably lead to the rise of TB [24, 25]. In addition, the increase in TB may be due to hemolysis caused by congestive liver [26]. Diminished hepatic blood flow can be caused by a compensatory increase in vascular resistance sparked by reduced cardiac output and mesenteric vasoconstriction by an activated renin–angiotensin system [27, 28]. When rats developed intestinal ischemia–reperfusion injury, portal venous blood flow to the liver decreased by 66% during ischemia and hepatic arterial blood flow decreased by 80% during reperfusion, resulting in transient acute liver dysfunction (decreased bile secretion, increased ALT, and reduced ATP in liver tissue) [29]. In a model of systemic hypoperfusion induced by cardiac tamponade in pigs, decreased aortic blood flow triggered depletion of the hepatic artery buffering response, leading to liver dysfunction without evidence of cellular damage [30]. At present, the possible reason for liver dysfunction covers hepatic venous congestion secondary to right ventricular dysfunction, ischemia attributed to reduced hepatic blood flow, and hypoxemia attributed to by hepatic artery hypoxia [31]. It is generally accepted that transaminase abnormalities were dominated by hepatocyte necrosis caused by ischemia and hypoxia, while cholestasis caused by hepatic congestion was accompanied by an increase in TB, but it is impractical to definitely distinguish between the two mechanisms, as they commonly coexist and mutually exacerbate the detrimental effects on the liver [31, 32]. ALF was not only observed in hypotensive patients, but also applied to the anoxic condition of the person with normal BP [33]. Furthermore, Rossello X et al. found that normal blood pressure did not equate to no hypoperfusion in acute HF, and patients with higher SBP were less likely to be accompanied by hypoperfusion [34]. Thus, it was reasonable for the patient with LI to have normal but lower SBP.

DCM is characterized by enlarged LVEDD and reduced LVEF, and its underlying molecular mechanism involves activation of the neurohumoral system, which forms the pathophysiological basis of LI occurrence [35, 36]. The effect of LI on prognosis of patients with DCM should be considered from AST, ALT and TB. Many studies have verified that AST, ALT and TB were associated with increased right atrial pressure, pulmonary wedge pressure, and central venous pressure, as well as decreased cardiac index [37–40]. This implied that the presence of LI may be a sign of tissue hypoperfusion or systemic congestion. Although proxies for congestion (i.e., NT-proBNP) and perfusion (i.e., SBP and LVEF) were incorporated into the final multivariate model, LI may represent hemodynamic changes that cannot be captured by traditional measures. Given that NT-proBNP is a serological marker for diagnosing HF and reflecting ventricular wall tension [41], patients with LI had higher NT-proBNP and may have more severe circulatory congestion. Therefore, it was not surprising that there was a higher proportion of NYHA IV in patients with LI. LI exhibited irreplaceability in terms of prognosis, which indicated that LI not only reflected the temporary alterations of hemodynamics of DCM, but also reflected the potential pathological state and disease development stage of DCM, affecting the long-term prognosis of DCM patients. Compared with invasive hemodynamic monitoring, liver function testing is convenient, inexpensive, and acceptable, which provides the possibility of repeated testing and clinical follow-up.

The protective effect of higher SBP was consistent with previous studies [42, 43], and the underlying mechanism may be that patients with higher SBP had better tissue perfusion and cardiac function. Meanwhile, NT-proBNP consistently presented prognostic value that cannot be ignored [44]. The negative effect of LVEDD in this study was not surprising, since it had been demonstrated that expansion of LVEDD increased the risk of HF and sudden cardiac death [45, 46]. Interestingly, LVEF was not included in the final model before PSM, which may be due to the interference of confounding factors. Nonetheless, LVEF remained an irreplaceable prognostic factor after PSM.

Vincenzo Nuzzi et al. found that permanent atrial fibrillation (AF) had a similar effect on the prognosis of DCM patients as LI in this paper, while paroxysmal or persistent AF presented neutral results [47]. However, the adverse effects of permanent AF were not evident in our study. The reasons may be as follows: on one hand, with the retrospective data, we cannot make a precise distinction between permanent, paroxysmal, and persistent AF. Consequently, studies mixing the above three may influenced the presentation of the results. On the other hand, whether patients underwent radiofrequency ablation after discharge and whether sinus rhythm was restored, which were not available and may also have interfered with the results. Kidney function included as a categorical variable (eGFR ≥ 60 mL/min/1.73 m^2^) in univariate COX analysis was not significantly associated with the prognosis of patients with DCM, which may be due to the exclusion of patients with severe chronic kidney disease. Furthermore, the use of RAS inhibitor may improve kidney function, thus affecting its prognostic effect on patients with DCM. More importantly, the vast majority of patients did not have chronic kidney disease and the decrease in eGFR may be a transient phenomenon.

As far as we know, the reverse action mechanism of cardiogenic LI on the heart may include the following three aspects. First, the development of LI may promote the release of inflammatory factors, activate cell signaling pathways, and cause myocardial dysfunction, similar to those found in cirrhotic cardiomyopathy [48]. Second, the possible changes in the metabolic function of hepatocytes alter the medication metabolism process and influence its efficacy [49]. Finally, the liver is a transit point for material metabolism, and LI may affect its energy supply to the heart and other vital organs [50].

We acknowledge that this report had some limitations. The general flaws of retrospective studies were also not immune to this study. First, in addition to liver function indicators, we had no additional liver morphological, pathological, and hemodynamic information to adequately assess the severity of LI and its impact on prognosis. Second, we did not analyze the duration and recovery of LI, and these data may have different effects on the results. Third, the absence of myocardial injury-specific markers prevented us from reliably ruling out the role of myocardial injury in the outcome. Fourth, the lack of data on the assessment of right heart function prevented us from considering all factors that might have influenced the results. Finally, variations in patient medication and device therapy were not recorded during follow-up. This may constitute a latent factor that interfered with the results, even if the baseline levels of drugs were balanced.

## Conclusions

The results of this study, which incorporated numerous patients with long-term follow-up, revealed that the occurrence of LI predicted a potentially bad prognosis in patients with DCM.

Attention to LI is beneficial to identify high-risk DCM patients and guide the next management.

## Supplementary Information

**Additional file 1: Table S1. **Mean dose of drug during hospitalization in the study population. **Table S2. **Cause of death in the study population. **Figure S1.** Kaplan–Meier curves of stratified analysis showed the occurrence of the primary outcome in patients with and without liver injury. (A) age ≤ 50 years, (B) age > 50 years, (C) male, (D) female.

## Data Availability

The supporting data can be acquired via the corresponding author on reasonable request.

## Abbreviations

ALF: Abnormal liver function
LI: Liver injury
ALB: Albumin
TB: Total bilirubin
AST: Aspartate transaminase
ALT: Alanine transaminase
HF: Heart failure
DCM: Dilated cardiomyopathy
LAD: Left atrium diameter
LVEDD: Left ventricular end-diastolic dimension
LVEF: Left ventricular ejection fraction
NYHA: New York Heart Association
PSM: Propensity score matching
SBP: Systolic blood pressure
BUN: Blood–urea–nitrogen
NT-proBNP: N-terminal pro-B-type natriuretic peptide
eGFR: Estimated glomerular filtration rate
RAS: Renin–angiotensin system
ACEI: Angiotensin-converting enzyme inhibitor
ARB: Angiotensin receptor blocker
ARNI: Angiotensin receptor neprilysin inhibitor
