# Supplementary material for: Prognostic potential of liver injury in patients with dilated cardiomyopathy: a retrospective study
Source: Eur J Med Res. 2022 Nov 8;27:237. doi: 10.1186/s40001-022-00876-9 (PMC9641949; doi:10.1186/s40001-022-00876-9)
Supplement: Supplementary file 1 — Additional file 1: Table S1. Mean dose of drug during hospitalization in the study population. Table S2. Cause of death in the study population. Figure S1. Kaplan–Meier curves of stratified analysis showed the occurrence of the primary outcome in patients with and without liver injury. (A) age ≤ 50 years, (B) age > 50 years, (C) male, (D) female. [file 40001_2022_876_MOESM1_ESM.docx]

**Table S1** The mean dose of drug during hospitalization in the study population

| Drug | Patients | Daily Dose(s) |
| --- | --- | --- |
| ACEI  Perindopril  Fosinopril  Benazepril  ARB  Irbesartan  Valsartan  Losartan  Telmisartan  ARNI  Sacubitril-valsartan  Beta-blocker  Metoprolol  Bisoprolol  Spironolactone  Digoxin | 362  3  7  28  4  1  3  52  410  61  501  415 | 3.10mg  5.00mg  10.71mg  133.93mg  80.00mg  50.00mg  66.67mg  81.25mg  42.26mg  2.99mg  23.83mg  148.19mg |

Metoprolol: Metoprolol succinate extended release

**Table S2** The cause of death in the study population

|  | Non-liver injury  (n=113) | Liver injury  (n=51) |
| --- | --- | --- |
| Cardiac cause  Stroke  Diabetic complication  Pneumonia  Lymphoma  Indeterminate cause | 74  3  1  2  0  33 | 39  2  0  0  1  9 |

**
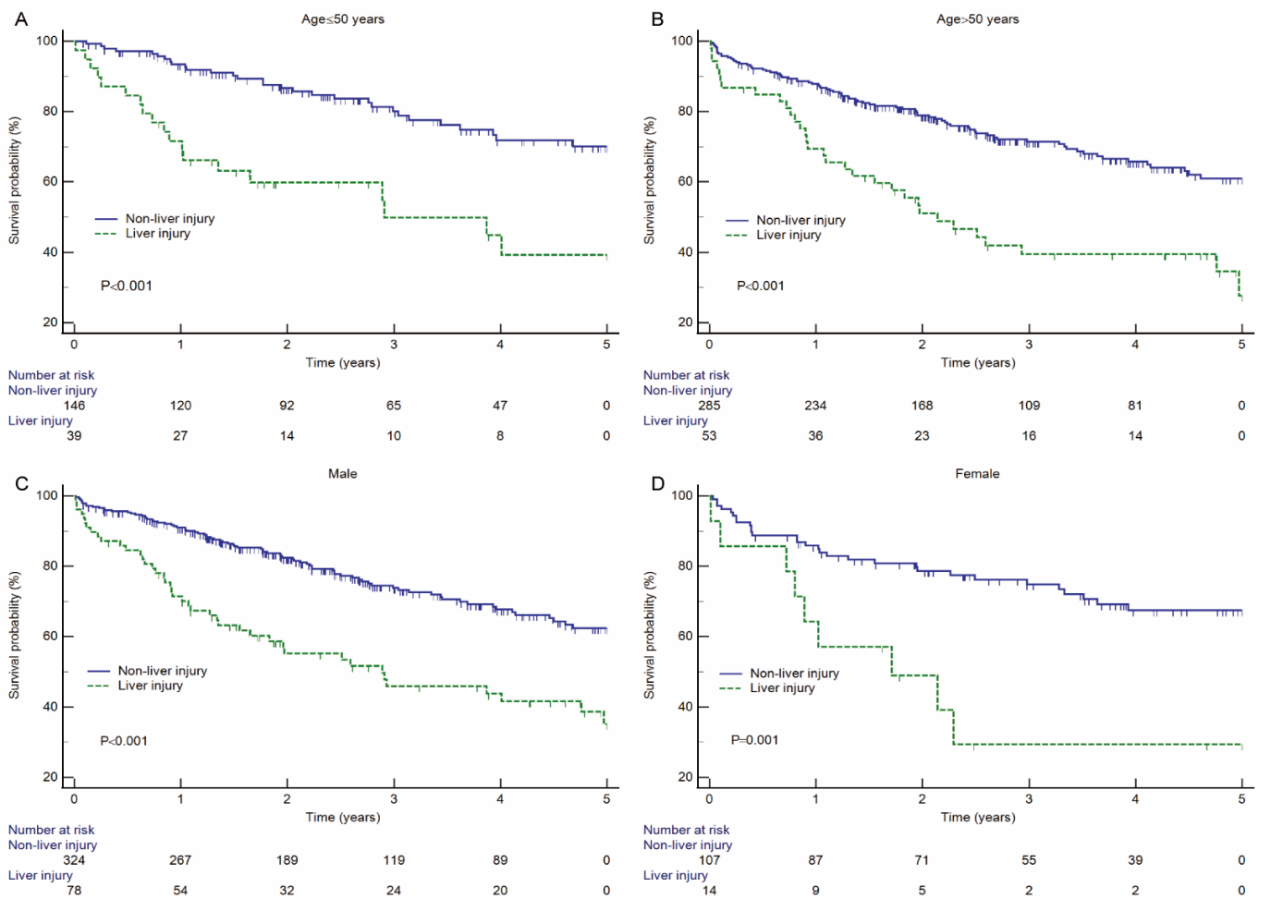
**

**Figure S1 Kaplan-Meier curves of stratified analysis showed the occurrence of the primary outcome in patients with and without liver injury. (A) age≤50 years, (B) age>50 years, (C) male, (D) female.**
